# Changes in propulsion technique and shoulder complex loading following low-intensity wheelchair practice in novices

**DOI:** 10.1371/journal.pone.0207291

**Published:** 2018-11-09

**Authors:** Marika T. Leving, Riemer J. K. Vegter, Wiebe H. K. de Vries, Sonja de Groot, Lucas H. V. van der Woude

**Affiliations:** 1 Center for Human Movement Sciences, University of Groningen, University Medical Center Groningen, Groningen, The Netherlands; 2 Swiss Paraplegic Research, Nottwil, Switzerland; 3 Amsterdam Rehabilitation Research Center | Reade, Amsterdam, The Netherlands; 4 Center for Rehabilitation, University of Groningen, University Medical Center Groningen, Groningen, The Netherlands; North Carolina State University, UNITED STATES

## Abstract

**Background:**

Up to 80% of wheelchair users are affected by shoulder pain. The Clinical Practice Guidelines for preservation of upper limb function following spinal cord injury suggest that using a proper wheelchair propulsion technique could minimize the shoulder injury risk. Yet, the exact relationship between the wheelchair propulsion technique and shoulder load is not well understood.

**Objective:**

This study aimed to examine the changes in shoulder loading accompanying the typical changes in propulsion technique following 80 min of low-intensity wheelchair practice distributed over 3 weeks.

**Methods:**

Seven able-bodied participants performed the pre- and the post-test and 56 min of visual feedback-based low-intensity wheelchair propulsion practice. Kinematics and kinetics of propulsion technique were recorded during the pre- and the post-test. A musculoskeletal model was used to calculate muscle force and glenohumeral reaction force.

**Results:**

Participants decreased push frequency (51→36 pushes/min, p = 0.04) and increased contact angle (68→94°, p = 0.02) between the pre- and the post-test. The excursion of the upper arm increased, approaching significance (297→342 mm, p = 0.06). Range of motion of the hand, trunk and shoulder remained unchanged. The mean glenohumeral reaction force per cycle decreased by 13%, approaching significance (268→232 N, p = 0.06).

**Conclusions:**

Despite homogenous changes in propulsion technique, the kinematic solution to the task varied among the participants. Participants exhibited two glenohumeral reaction force distribution patterns: 1) Two individuals developed high force at the onset of the push, leading to increased peak and mean glenohumeral forces 2) Five individuals distributed the force more evenly over the cycle, lowering both peak and mean glenohumeral forces.

## Introduction

People who lose lower-limb function need to rely on their arms to maintain mobility and learn to propel a wheelchair in the early stages of rehabilitation. The anatomy of the upper-extremities, specifically the relatively small muscle mass and high glenohumeral joint mobility, makes the shoulder complex vulnerable to overuse injuries. Because of the low physical capacity of persons at the beginning of rehabilitation and the highly straining character of wheelchair propulsion, shoulder pain frequently develops at this stage [1]. Among the individuals who developed shoulder complaints during early rehabilitation, only 20% will show any improvement over time [2].

The Clinical Practice Guidelines for preservation of upper limb function following spinal cord injury describe various ways to minimize the shoulder injury risk, such as: proper wheelchair configuration or assistance during transfers but also using a proper wheelchair propulsion technique [3]. Shoulder load and propulsion technique are thought to be linked as wheelchair propulsion is a highly repetitive task, where the same motion is performed approx. 2700 times per day [1,4]. This is especially evident in novice wheelchair users as their propulsion is characterized by a very high frequency, high peak forces and small contact angle of the hand on the handrim [5–7]. In contrast, the advice of The Clinical Practice Guidelines is to minimize the peak forces and push frequency during propulsion and maximize the contact angle of the hand with the handrim.

We know from a number of studies that as natural motor learning progresses wheelchair users naturally improve their technique i.e. lower the push frequency and increase the contact angle [5,6,8–11]. Even though the Clinical Practice Guideline states that this should be a more optimal technique, experimental evidence supporting this advice is missing. It is unclear how the changes in propulsion technique taking place during practice relate to the mean and peak loads on the shoulder. That is because the propulsion technique as defined in this study, includes spatio-temporal variables which are calculated based on the kinetic data measured on the level of the forces applied to the handrim by the user. To gain understanding of what the association between the propulsion technique and the local strain on the shoulder complex is, a combination of modeling, kinematics and kinetics needs to be implemented.

To do that, we will use data from a previously published study, which found typical improvements in propulsion technique following low-intensity wheelchair practice, specifically a large increase in contact angle and decrease in push frequency [6]. The goal of the current study will be to investigate the association between the typical changes in propulsion technique and the shoulder load. The effect of intervention will be omitted as it was already described [6]. The focus will be laid on the description of the co-emergence of the typical improvements in propulsion technique observed during a learning process and changes in the local load on the shoulder. So far, this has only been investigated in the very initial stages of practice. Vegter et al. [5] found a reduced push frequency and larger contact angle following a 12 min practice period. Interestingly, the improvements in the propulsion technique were concomitant with higher shoulder load expressed as an increase in relative muscle forces, especially within the rotator cuff muscles and higher peak and mean glenohumeral reaction forces [5]. The effects of practice on the local strain on the shoulder exceeding a 12-min period are unknown.

The goal of this study is to describe the changes in shoulder load and kinematic characteristics of movement taking place after the 80-min low-intensity wheelchair practice. To be able to exclude the effect of experience and secondary conditions on the outcomes, a group of able-bodied participants with no wheelchair experience was selected for this study. We hypothesize that a decrease in push frequency and increase in contact angle will contribute to a decrease in mean glenohumeral reaction force and individual muscle forces. Identifying properties of the propulsion technique that are beneficial for the shoulders could be used in the future to propose evidence-based interventions to target early prevention of shoulder pain in manual wheelchair users.

## Materials and methods

### Participants and ethics statement

A convenience sample consisting of seven able-bodied men (Age median = 23 years, interquartile range (IQR) = 5 years; Body mass median = 76 kg, IQR = 19 kg) participated in the study. This is a subsample of 17 participants who were included in the previous study [6]. Inclusion of only 7 participants from the original sample is a consequence of the technical difficulties during the very complex data collection process. All participants provided written informed consent following detailed information about the character of the study. The protocol of the study was approved by the Local Ethics Committee, of the Center for Human Movement Sciences, University Medical Center Groningen, University of Groningen, The Netherlands. Individuals were eligible for inclusion if they had no previous experience with wheelchair propulsion and no severe musculoskeletal complaints, especially within the upper extremities and the trunk.

### Study design

**Fig 1:**
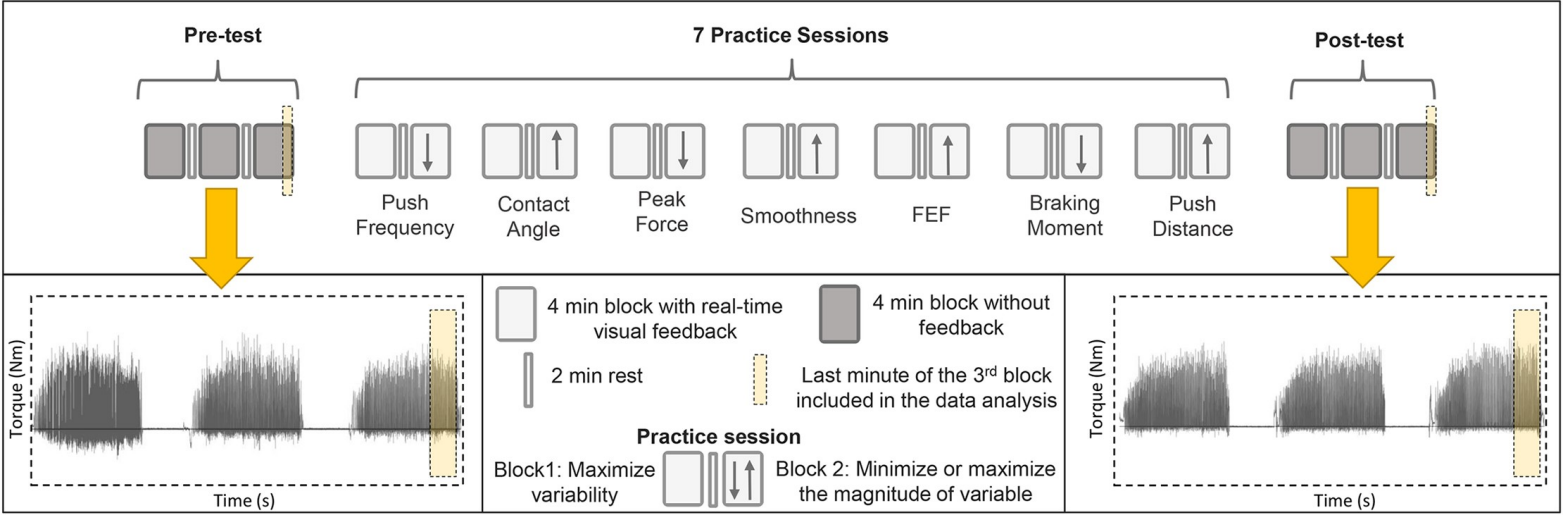
All participants (N = 7) performed the pre- and the post-test and seven practice sessions in between. The pre-test and the post-test consisted of 3 blocks (4 min each) of propulsion during which participants received no feedback and the practice sessions consisted of 2 blocks (4 min each) during which real-time visual feedback on a specific propulsion technique variable was given.

All participants received 80-min of handrim wheelchair practice at a submaximal intensity distributed across 3 weeks (Fig 1). The 80-min experiment consisted of a pre- and post-test (3x4 min exercise blocks each with 2 min break between the blocks) and 7 practice sessions (2x4 min exercise blocks each with 2 min break between the blocks) between the pre- and the post-test. During the pre- and the post-test participants received no feedback or instruction. During the 7 practice sessions participants received real-time visual feedback on their propulsion technique. At each session, a different propulsion technique variable was presented. Each variable was presented only once, meaning that there were 7 unique propulsion technique variables: push frequency, braking moment, contact angle, peak force, push distance, smoothness and fraction effective force (Table 1). Participants were instructed to be as variable as possible (applying stroke to stroke variation in a given propulsion technique variable e.g. alternating short and long pushes or applying variable peak forces) in the first 4 min block of practice and to optimize the variable in the prescribed direction in the second 4 min block. The name and the description of the practiced variable were not provided to encourage motor exploration. Therefore the name of the variable on the screen was covered. The last minute from the pre- and last minute from the post-test were compared to determine the change in propulsion technique and shoulder load following low-intensity practice. The last minute from the pre-test was chosen because changes in the propulsion technique and shoulder load for the first 12 minutes of practice in able-bodied individuals have been documented in a previous study [5].

**Table 1:** Propulsion technique variables.

Propulsion variable (unit)	Description	Equation
Push frequency (push/min)	The number of pushes performed during one minute	N_pushes_/Δt
Braking moment (Nm)	The braking moment applied to the handrim with each push. The sum of braking moment exerted on the handrim during coupling and decoupling of the hand	Σ_end_(i)_:start_(i+1) (Tz · ΔØ)
Contact Angle (°)	The angle measured along the handrim, where subject's hand maintained contact with the handrim during each push	Ø_end_(i)_-_Ø_start_(i)
Smoothness	The ratio of mean to peak force per push	Mean_(start:end)_ (Fx^2^+ Fy^2^+ Fz^2^)^0,5^/Max_(start:end)_ (Fx_2_+ Fy_2_+ Fz_2_)^0,5^
FEF (%)	The ratio of effective to total force that was applied to the handrim during one push	Mean_(start:end)(_((Tz/r)/((Fx^2^+ Fy^2^+ Fz^2^)^0,5^))·100%
Push distance (m)	The distance covered with each push	Mean_(start:end)_V·Δt
Peak force (N)	3d peak force applied to the handrim during one push	Max_(start:end)_ (Fx^2^+ Fy^2^+ Fz^2^)^0,5^
Positive work per push (J)	The power integrated over the Contact angle of the push.	∑start : end(Tz * ΔØ)
GH start position (mm)	Horizontal position of the glenohumeral joint (GHx) at the start of the push with respect to the wheel-axle (WAx)	GHx_start_(i) ‐ WAx_start_(i)
AL displacement (mm)	The position difference between AL at the start and end of the push phase. AL represents M5, EM, GH, IJ	AL_end_(i) ‐ AL_start_(i)

First seven variables were used in a form of visual feedback during the practice sessions. Frequency, contact angle, peak force, positive work per push and displacement of anatomical landmarks were used as outcome variables to compare the change in shoulder load between the pre- and the post-test. All variables except cadence were calculated as an average value of all pushes performed during the last minute of the last practice block. Equations from Vegter et al.[9]; table from Leving et al.[6]

Abbreviations: t, time(s); start(i), start of the current push (sample); end(i), end of the current push (sample); Tz, torque around wheel axle (Nm); Ø, angle (rad); Fx, Fy and Fz, force components (N); r, wheel radius (m); V, velocity (m/s); AL, Anatomical Landmark; M5, Fifth metacarpal; EM, Medial epicondyle of humerus; GH, Glenohumeral joint; IJ, Suprasternal notch

### Experimental setup

**Fig 2:**
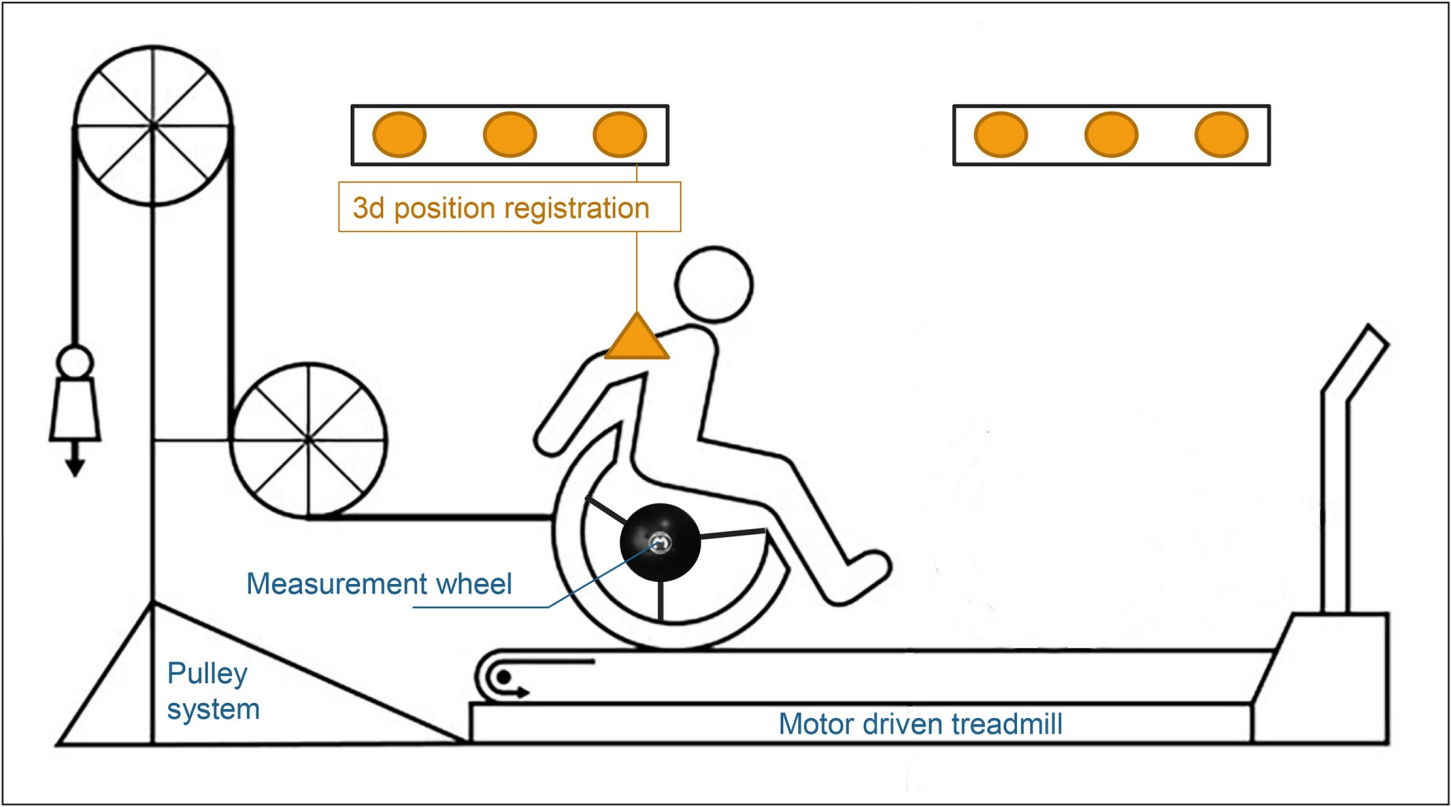
Experimental setup. Participants propelled a wheelchair on a motor-driven treadmill during all testing and practice sessions at a constant velocity of 1.11 m/s and relative power output of 0.24 W/kg. Power output was imposed using a pulley system. Kinetics and kinematics of propulsion were recorded during the pre- and the post-test. Modified figure from Vegter et al [5].

The 80 min experiment was performed by each participant in the same experimental handrim wheelchair with 24 inch wheels, 5° camber, seat height of 0.54 m and seat width of 0.45 m (Double Performance BV, Gouda,The Netherlands) placed on a level motor-driven treadmill (length x width = 2.4 m x 1.2 m; Forcelink b.v, Culemborg The Netherlands) (Fig 2). Tire pressure of the rear wheels was set at 600 kPa during all practice and test sessions. Treadmill velocity of 1.11 m/s and power output of 0.24 W/kg body mass were maintained throughout the experiment. Required power output was imposed using a pulley system. The mass of the pulley was determined individually for each participant based on the results of the wheelchair drag test which took place prior to the experiment [12,13].

### Propulsion technique and kinematics of wheelchair propulsion

Kinetics and kinematics of wheelchair propulsion were recorded continuously during the pre- and the post-test. The data of the last minute of the pre-test was compared with the data of the last minute of the post-test to examine the changes in shoulder loading following the low-intensity wheelchair propulsion practice.

#### Propulsion technique

Software of the instrumented 24˝ Optipush wheel (MAX Mobility, LLC, Antioch, TN, USA), which measures 3D forces and torques applied to the handrim, was used to gather data from the right wheel and to provide the real-time visual feedback. The measurement frequency of Optipush wheel was set at 200 Hz. The output from the measurement wheels was analyzed using custom-written Matlab algorithms [9] (Table 1).

#### Kinematics

**Fig 3:**
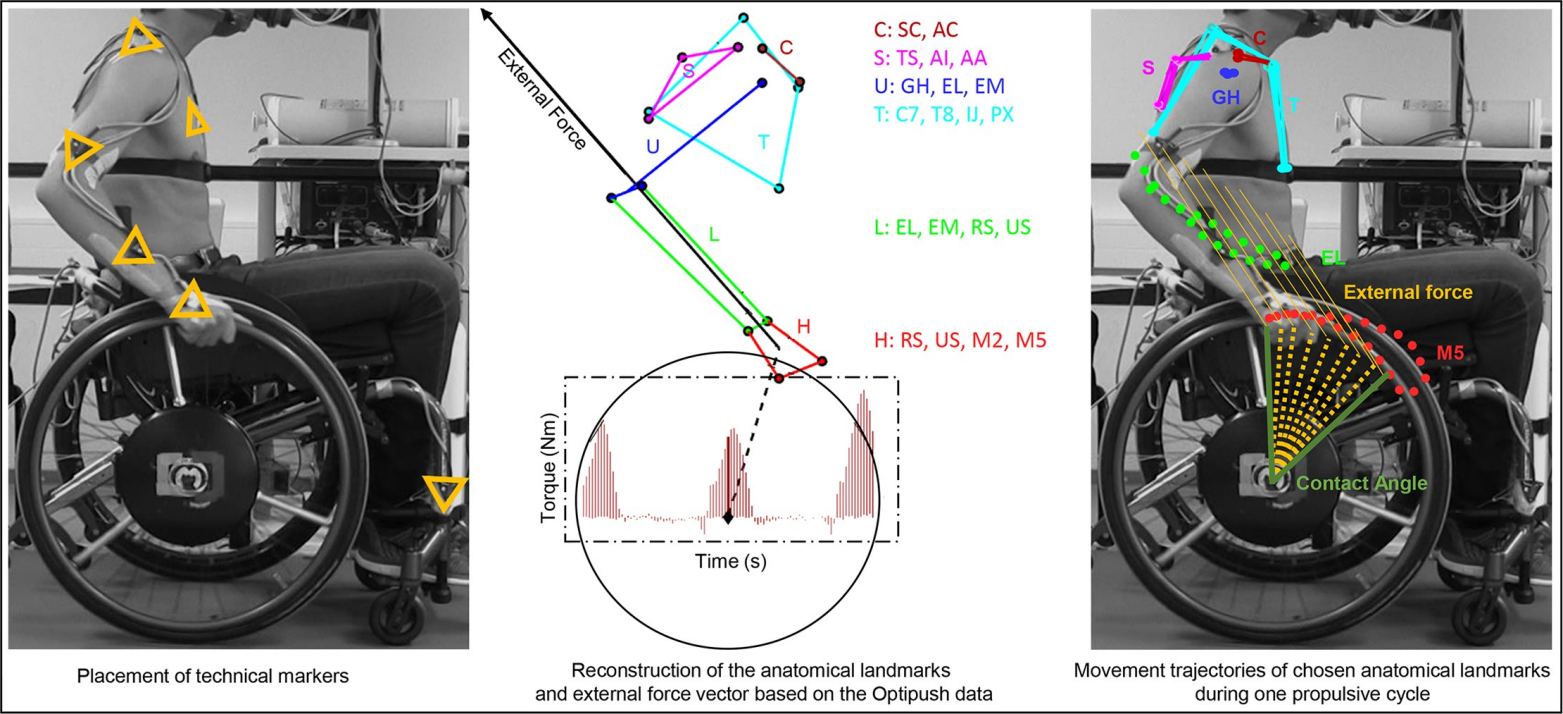
Typical placement of the technical markers (left panel), reconstruction of the anatomical landmarks (middle) and movement trajectories of the scapula, trunk, clavicle, EM and M5 anatomical landmarks combined with the external force vector exerted during one exemplary propulsive cycle (right panel). Abbreviations: M5, Fifth metacarpal; EM, Medial epicondyle of humerus; GH, Glenohumeral joint; IJ, Suprasternal notch; C, Clavicle; S, Scapula; U, Upper arm; T, Trunk; L, Lower arm; H, Hand; SC, sternoclavicular joint; AC, acromioclavicular joint; TS, trigonum spinae scapulae; AI, Angulus Inferior Scapulae; EL, Lateralepicondyle of humerus; C7, Processus spinosus of 7^th^ cervical vertebra, T8, Processus spinosus of 8^th^ thoracic vertebra; PX, Processus xiphoideus; RS, Radial styloid; US, Ulnar styloid; M2, Second metacarpal.

Kinematic data were collected using an optoelectronic camera system (Optotrak, Northern Digital, Waterloo, Canada) at 100Hz with technical cluster markers attached to the right side of the participant’s body and to the rigid frame of the wheelchair (Fig 3). The location of anatomical landmarks was determined in relation to their technical clusters. Based on this calibration procedure, the positions of the anatomical landmarks were reconstructed for each participant (Fig 3) and used to create the joint coordinate systems of the shoulder, elbow and wrist [14]. The location of the rotation center of the glenohumeral joint was calculated using the regression method proposed by Meskers et al. [15].

### Delft shoulder and elbow model

To evaluate the load on the shoulder complex during wheelchair propulsion, inverse dynamics can be used as input for a musculoskeletal model to estimate joint reaction forces and individual muscle force. The model used in this study was the Delft Shoulder and Elbow Model (DSEM). The DSEM is a finite-element 3D inverse-dynamics model consisting of 22 muscles (31 muscle parts), divided into 155 muscle elements [16]. It includes all bones and joints of the shoulder and has 17 degrees of freedom of which six for the thorax are the moving base. Bones are modelled as rigid bodies, muscles as active trusses and ligaments as passive trusses. Information concerning muscle architecture parameters was obtained from cadaver studies and was therefore not individualized for each participant [17,18]. This model was used previously to estimate the shoulder load during handrim wheelchair propulsion [5,19–21]. Kinematic input for the model consisted of the orientations of the humerus, scapula, thorax, forearm and hand and the position of the jugular notch (incisura jugularis). Kinetic input consisted of the 3D external forces applied by the hand of the user to the handrim. Based on the recorded motions and external loads, the muscle and joint contact forces are calculated as model outputs through an inverse-dynamics analysis (Table 2). DSEM allows two optimization methods: static and dynamic optimization [22]. In this study, dynamic optimization was used since it takes the dynamics of segments and muscles in account. An energy-related cost function, with the objective to minimize the summed energy consumption, was used to estimate the individual muscle forces: $\sum_{i=1}^{n}{\dot{E}}_{\mathrm{mi}}=\sum_{i=1}^{n}{\dot{E}}_{\mathrm{fi}}+{\dot{E}}_{\mathrm{ai}}$ [23]. In this function the muscle energy consumption (E_m_), is based on the two major energy-consuming processes in the muscle: detachment of cross bridges (E_f_) and re-uptake of calcium (E_a_). The relative muscle forces were calculated to be able to compare the contributions between muscles taking their physiological cross-sectional area into account. Relative muscle forces were calculated as a percentage of the maximum force based on a force per physiological cross-sectional area of 100 N/cm^2^ [17]. Five consecutive pushes of the final minute of the pre- and the post-test were inserted in the model. The selected pushes were assumed to be representative of the pushes within the given minute. Therefore, they were chosen based on their mean power output per push, which could not differ more than 10% from the mean power output calculated for all pushes within the last minute of propulsion. The outcome measures are calculated either per push or per cycle. Propulsive cycle is defined in accordance with previous literature and consists of the push and the recovery phase [24]. The push begins with the initial hand contact and ends with the release of the hand from the handrim. The recovery phase is the period between the two consecutive pushes.

**Table 2:** DSEM outcome variables.

Variable (unit)	Description
GH mean Net Moment/Push (Nm)	The mean external net moment of the reaction force around the glenohumeral joint
GH peak Net Moment/Push (Nm)	The peak external net moment of the reaction force around the glenohumeral joint
HU mean Net Moment/Push (Nm)	The mean external net moment of the reaction force around the Humeroulnar joint
HU peak Net Moment/Push (Nm)	The peak external net moment of the reaction force around the Humeroulnar joint
Muscle Power total mean/Push (W)^a^	The mean sum of all muscle powers during the push
Muscle Power total peak/Push (W)	The peak sum of all muscle powers during the push
Muscle Power total mean/Cycle (W)	The mean muscle power performed per cycle
GH Reaction force mean/Push (N)	The mean glenohumeral reaction force per push
GH Reaction force peak/Push (N)	The peak glenohumeral reaction force per push
GH Reaction force mean/Cycle (N)	The mean glenohumeral reaction force per cycle

Table from Vegter et al.[5]

^a^ Muscle power is calculated per a contractile element by multiplying the estimated force of each element with its shortening velocity.

### Statistical analysis

Based on the number of participants N = 7, non-parametric testing was chosen. Unless reported otherwise a median and interquartile (IQR) range are reported to describe the outcomes. To evaluate the change in kinetics, kinematics and shoulder load between the last block of the pre-test and the last block of the post-test the Wilcoxon Signed Rank test was used. Significance level of p<0.05 was used. The variables compared with the Wilcoxon Signed Rank test were: propulsion technique variables (frequency, contact angle, peak force and net work per push), 3D displacement of anatomical landmarks, mean and peak net moments per push around the glenohumeral and humeroulnar joints and mean and peak muscle powers and glenohumeral joint reaction forces. The effect size of the Wilcoxon Signed Rank test was calculated using the following formula $r=\frac{Z}{\sqrt{N}}$, where Z is the test statistic and N, the total number of observations [25]. Effect sizes >0.1 are considered small, >0.3 moderate and >0.5 are considered large [26].

## Results

All participants (N = 7) completed all practice and test sessions. The relative power output did not change significantly between the pre- and the post-test (0.249 W/kg → 0.234 W/kg, p = 0.18). Results of all statistical analyses are presented in Table 3.

**Table 3:** Outcomes (Median (IQR)) for all participants (N  =  7) for the last minute of the third practice block of the pre- and the post-test and outcomes of statistical analyses (Wilcoxon Signed Rank Test: p<0.05) including the effect size.

		Median (Interquartile Range)				
Kinetics		PRE	POST	p value	Relative change	Effect size
	Relative Power Output (W/kg)	0.249 (0.01)	0.234 (0.03)	0.18	6%	-0.36
	Frequency (push/min)	51 (27)	36 (22)	0.04	29%	-0.54
	Contact Angle (°)	68 (14)	94 (24)	0.02	38%	-0.63
	Positive work per push (J)	12 (4)	14 (5)	0.06	17%	-0.50
	Peak Force (N)	73 (19)	82 (20)	0.40	12%	-0.23
Kinematics						
	M5 displacement (mm)^a^	347 (134)	350 (80)	0.24	1%	-0.32
	EM displacement (mm)^a^	297 (64)	342 (56)	0.06	15%	-0.50
	GH displacement (mm)^a^	37 (24)	27 (66)	0.40	27%	-0.23
	IJ displacement (mm)^a^	27 (20)	31 (30)	1.00	15%	0
	GH start position (mm)	-62 (51)	-46 (100)	0.74	26%	-0.09
Net moments per push						
	GH mean Net Moment (Nm)	14 (5)	16 (4)	0.40	14%	-0.23
	GH peak Net Moment (Nm)	31 (13)	33 (6)	0.61	6%	-0.14
	HU mean Net Moment (Nm)	4 (4)	1 (5)	0.13	75%	-0.41
	HU peak Net Moment (Nm)	16 (8)	15 (2)	0.61	6%	-0.14
Model results						
	Muscle power total mean per push (W)	62 (15)	56 (44)	0.87	10%	-0.05
	Muscle power total peak per push (W)	138 (140)	107 (158)	0.74	22%	-0.09
	Muscle power total mean per cycle (W)	37 (6)	28 (9)	0.18	24%	-0.36
	GH Reaction force mean per push (N)	392 (208)	448 (175)	1.00	14%	0
	GH Reaction force peak per push (N)	889 (353)	920 (323)	0.87	3%	-0.05
	GH Reaction force mean per cycle (N)	268 (105)	232 (49)	0.06	13%	-0.50

^a^ 3D displacement of anatomical landmarks during push phase

Abbreviations: M5, Fifth metacarpal; EM, Medial epicondyle of humerus; GH, Glenohumeral joint; IJ, Suprasternal notch; HU, Humeroulnar joint

### Effect of practice on propulsion technique and kinematics of propulsion

**Fig 4:**
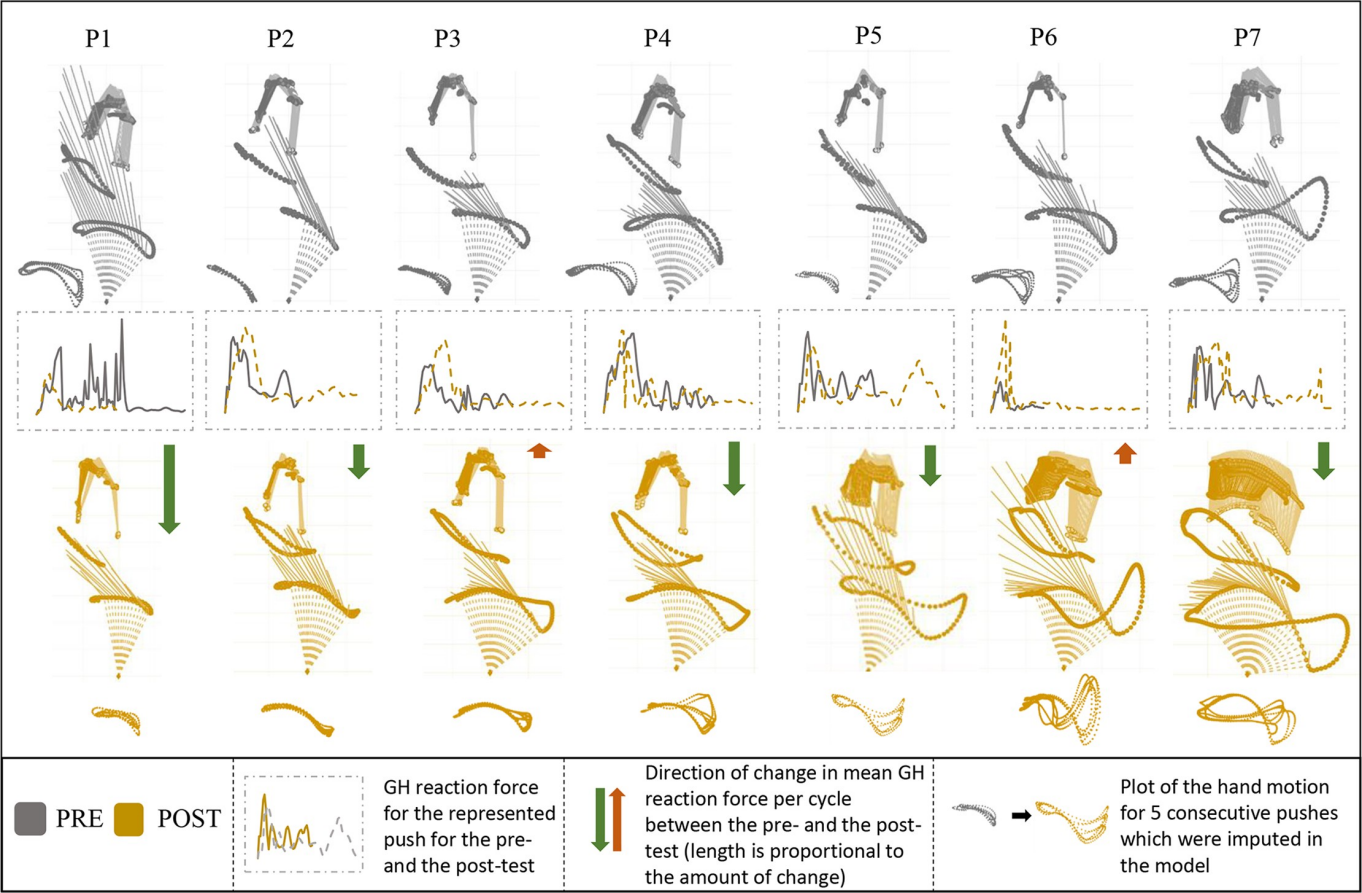
**Typical example of one propulsive cycle for each participant (P1-P7) for the pre- (top) and the post-test (bottom section).** The kinematic and kinetic input (reaction force vector) for the model in relation to the trajectories of the shoulder, elbow and hand over the push and recovery phase (cycle) for the pre- (grey) and the post-test (yellow) (row 1 and 3). Glenohumeral reaction force for the presented push for the pre- and the post-test (row 2). The illustrations of participants were sorted in ascending order based on the hand excursion at the post-test.

The change in kinetic and kinematic aspects of movement during one typical push for the pre- and the post-test for each participant is illustrated in Fig 4.

### Propulsion technique

Push frequency decreased significantly between the pre- and the post-test (51 push/min → 36 push/min, p = 0.04), while the contact angle increased (68.9° → 94.5°, p = 0.02). Positive work approached significance (p = 0.06) and peak force per push (0.40) did not change significantly between the pre- and the post-test. The effect size was large for frequency and contact angle and moderate for positive work per push and peak force.

### Kinematics

To describe the change in kinematic aspects of movement, the displacement of anatomical landmarks was used. On the level of the group the displacement of the fifth metacarpal (M5), medial epicondyle of humerus (EM), glenohumeral joint (GH), suprasternal notch (IJ) did not change significantly between the pre- and the post-test. The excursion of the upper arm increased in 6 out of 7 participants (humeral medial epicondyle displacement, 297 mm → 342 mm, p = 0.06), approaching significance, while excursion of the hand, trunk and shoulder did not change between the pre- and the post-test.

### Effect of practice on shoulder complex loading

The mean and peak net moments of the external force around the glenohumeral and humeroulnar joints did not change significantly between the pre- and the post-test (Table 3). The mean glenohumeral reaction force per cycle decreased between the pre- and the post-test in 5 out of 7 participants, approaching significance in the whole group (269 N → 233 N, p = 0.06). Effect size of the decrease in the mean GH reaction force per cycle was large, r = 0.5. Mean and peak glenohumeral reaction force per push did not change significantly and exhibited low effect sizes. Mean muscle power per cycle decreased in 6 out of 7 participants but the group means were not significantly different (37 W→  28 W, p = 0.18). Effect size of change in mean muscle power per cycle was moderate r = 0.36. Change in peak and mean muscle power per push was not significant and had a low effect size.

### Effect of practice on individual muscle force

**Fig 5:**
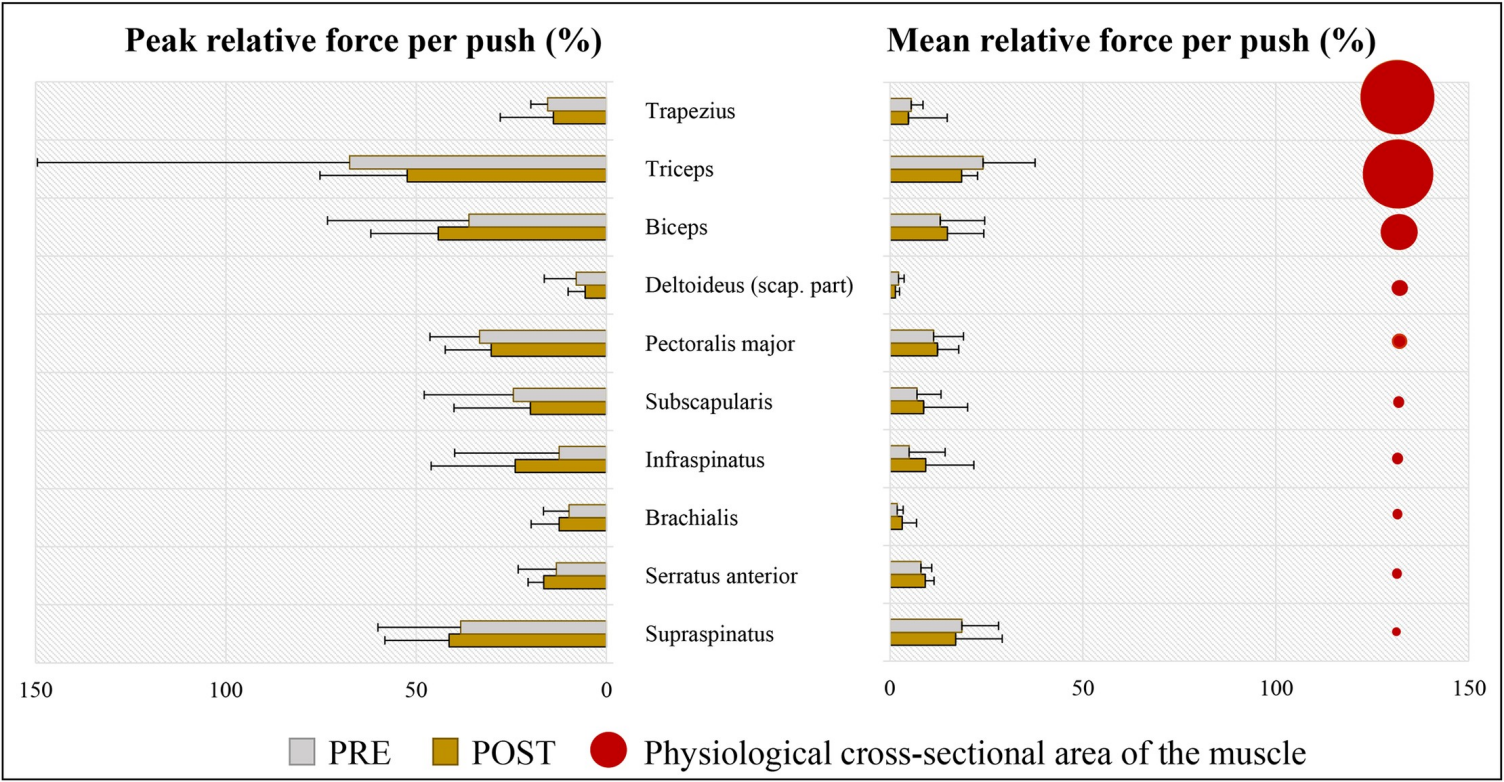
Relative peak and mean forces (N = 7) of individual muscles during the push phase for the pre- and the post-test. **The proportional differences in the physiological cross-sectional area between the muscles are shown (right).** Bars represent the median and the error bars represent the IQR. Only the muscles that had mean muscle forces during the push phase larger than 25 N are illustrated. The muscles are arranged in a descending order of physiological cross-sectional area.

The contribution of individual muscles relative to their theoretical maximal force (Fig 5), as opposed to the absolute muscle force, takes the differences in size between the muscles into account. The higher the relative muscle force, the bigger the chance of injury as higher numbers mean that a muscle approaches its maximum force generating capacity. The highest mean relative forces during the push phase at the pre- and the post-test were found in triceps (24.1% → 18.6%), followed by one of the rotator cuff muscles: supraspinatus (18.6% → 17.1%). Also biceps (13.0% → 14.9%) and pectoralis major (11.3% → 12.3%) developed quite high relative muscle forces. The same muscles also needed to endure the highest peak relative forces in the push phase. Yet, none of the changes in relative muscle force between the pre- and the post-test were significant.

**Fig 6:**
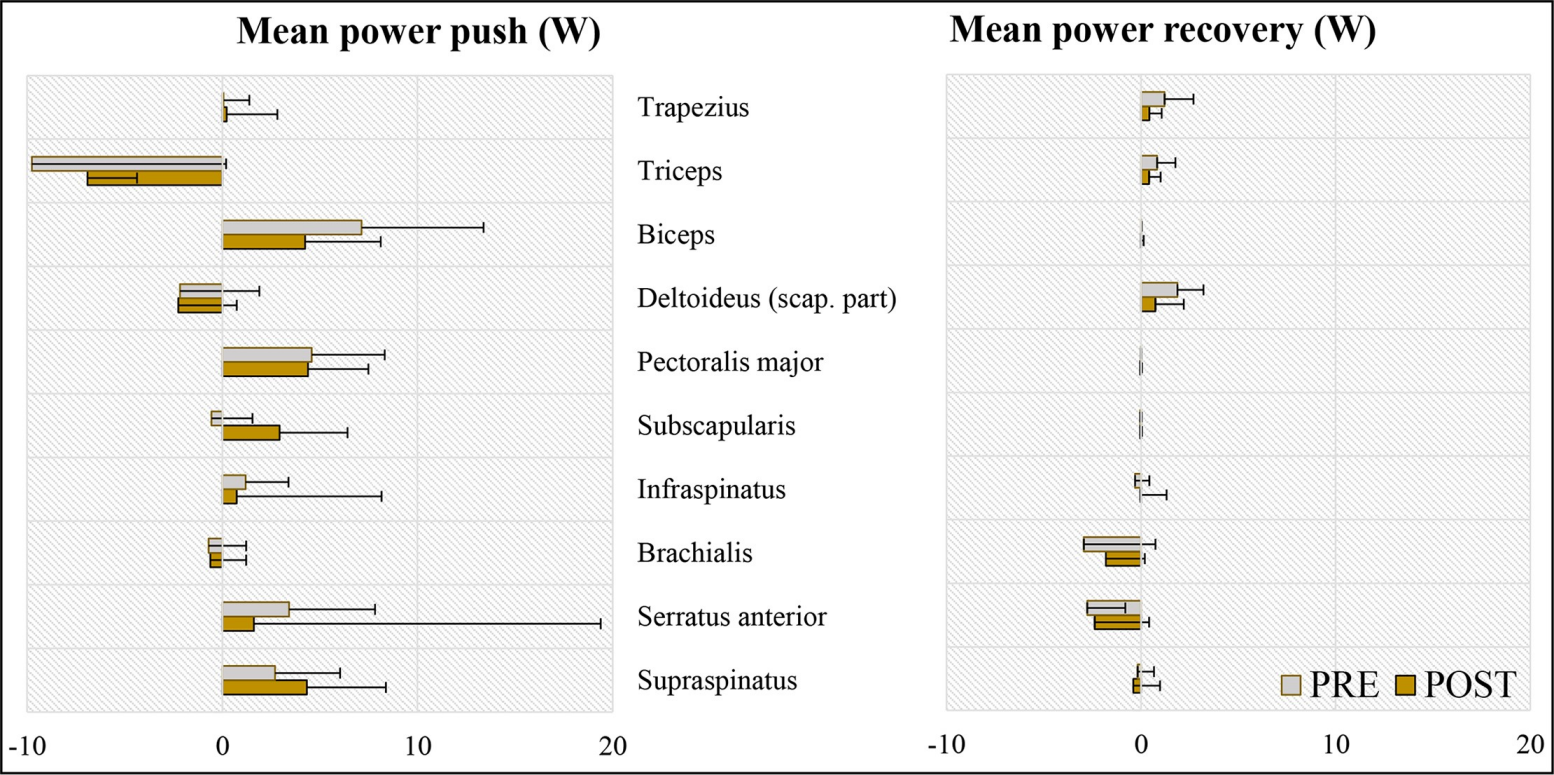
**DSEM estimates of individual muscle powers (N  =  7; median and IQR) during the push (left) and recovery (right) phase for the pre- and the post-test.** Only the muscles that had mean muscle forces during the push phase larger than 25 N are illustrated. The muscles are arranged in a descending order based on their physiological cross-sectional area.

Triceps delivered the highest mean negative power contribution (-9.8 W → -6.9 W) and biceps delivered the highest mean positive contribution (7.1 W → 4.2 W) during the push phase at both testing occasions (Fig 6). Although the mean power during the push did not change significantly over time, there is a decreasing trend visible for all muscles except the supraspinatus, subscapularis and deltoideus in the push phase and supraspinatus in the recovery phase.

## Discussion

The goal of this study was to examine the changes in upper-limb dynamics and shoulder complex loading accompanying the typical changes in propulsion technique following 80 min of low-intensity handrim wheelchair propulsion practice. This study is unique, as it looked at changes in shoulder loading over three weeks in a group of inexperienced able-bodied individuals. Finding comparable studies is therefore challenging. In order to allow the reader a proper interpretation of the results, we put our findings in light of both able-bodied and actual wheelchair user literature, taking into consider the differences between study protocols and participants.

### Propulsion technique

The changes in the propulsion technique, based on the forces and moments applied to the handrim, found in the current study are very similar in direction and magnitude to the changes reported by other low-intensity practice studies in able-bodied individuals [7,10,11,27]. For instance the change in push frequency in the current study from 51 to 36 pushes/min matches the direction and magnitude of change in the study of de Groot (61 → 42 push/min) [27] or in the study of Vegter (68 → 49 push/min) [10]. Both studies included able-bodied individuals who received a similar dose of practice as the current study. When considering the values of push frequency and contact angle at the post-test, they closely resemble the values of those parameters in experienced wheelchair users, e.g. contact angle of 94° in the current study vs 98° in the study with 59 experienced wheelchair users with paraplegia [24], or 97° in an experiment including 21 experienced wheelchair users [28], frequency of 36 pushes/min here vs 43 pushes/min in a study which included 10 adolescent experienced wheelchair users with various disabilities [29].

### Kinematics

Despite the rather uniform changes in propulsion technique, participants developed various kinematic solutions in response to an identical task (Fig 4). The only change that was consistent across the participants was the increase in the excursion of the upper arm (Medial Epicondyle). Involvement of the trunk during propulsion varied strongly among the participants. The Suprasternal Notch displacement increased in 3 participants and did not change in the other 4. Change in the excursion of the hand also varied in magnitude and direction among the participants with 5 participants increasing and 2 decreasing it (Fig 4). There was no apparent pattern between the excursion of various landmarks and change in mean and peak GH forces. Meaning that participants who managed to lower the shoulder load between the pre- and the post-test developed rather varying kinematic solutions. Wheelchair propulsion is a redundant task, which means that there are more motor components involved in the production of action than are essentially required. In other words, the same forces can be applied to the handrim using various upper-arm and trunk configurations. This may explain why the users developed various kinematic patterns without compromising the equivalent outcome [30], the production of a mean velocity and power output over time.

### Effect of practice on shoulder complex loading

In contrast to the rather variable kinematic output, it seems that participants exhibited two glenohumeral reaction force distribution patterns during the propulsive cycle. Two participants (P3 and P6 in Fig 4) applied high force in a rapid and relatively short-lasting push, which led to a high increase in the peak GH force during the push and, as a consequence, to an increase in mean GH force per cycle. All other participants managed to maintain the treadmill velocity by distributing the force more evenly over the propulsive cycle and in consequence decreasing the peak GH forces. This, combined with lowering the push frequency, led to a decrease in mean GH force during the propulsive cycle.

Next to the force distribution pattern, there may be another factor that caused an increase in shoulder loading in P3 and P6. A Recent study by Requejo et al. [31] concluded that a more anterior placement of the hand at release relates to a higher shoulder load. As visible in Fig 4, at the post-test, P3, P6 and P7 had the most anterior placement of the hand when decoupling from the handrim. From those three participants, P7 was the only one who lowered the shoulder load at the post-test. This could be related to the fact that P7 distributed the handrim forces evenly throughout the push, avoiding high peak forces when the hand was in the vulnerable anterior position. However we would like to emphasize that based on the findings of this and previous experiment [31], we cannot explain what the exact factors are that cause the anterior hand placement at release to be potentially more harming for the shoulder. We recommend to investigate this issue in the future.

The median peak GH forces of 920 N found at the post-test are comparable to those reported in other wheelchair propulsion studies with experienced wheelchair users [1,5,32] and able-bodied participants [5,33]. However it should be noted that P6 increased the peak GH force at the post-test to over 2300 N per push, achieving a value, almost 1300 N (~60%) higher than the second highest score recorded in the current study. This exceptionally high value is comparable to peak forces measured during ramp propulsion (2555 N) [32]. Peak force of P6 measured at the handrim was nearly 30% higher than the second highest peak force recorded at the post-test. High peak force was necessary to maintain the belt velocity as P6 developed the lowest push frequency recorded at the post-test of 21 pushes per minute. The peak forces in the majority of participants are lower than peak forces found during for example weight-relief lifting in a wheelchair (~1500 N) [34,35]. However it should be considered that wheelchair propulsion is a highly repetitive task, meaning that the cumulative tissue overload can be much higher than for weight-relief lifting.

Maintaining a certain velocity and power output during wheelchair propulsion can be achieved in a number of ways, keeping in mind that there is a relationship between the propulsion technique variables. As push frequency decreases during practice, the contact angle and work per push need to increase to maintain a constant velocity. The advice of current clinical practice guidelines [3] to use long strokes and low frequency during propulsion, is based on the assumption that larger contact angle would lead to a decrease in the peak glenohumeral reaction forces, as load is evenly distributed over a larger angle. However, there may be a threshold beyond which excessive lowering in frequency, despite the high contact angle, is not desired as it has to be compensated by an increase in peak forces, in order to maintain constant velocity, like in the example of P6. This statement is supported by the study of Rankin [36], which suggested that altering push frequency or contact angle to extreme values is less effective in lowering overall muscle demand than moderate adjustments in technique. Similar statements were made for other propulsion technique variables such as peak force [36] or fraction effective force [37].

Although assumed critical, it is not clear whether the goal of wheelchair propulsion practice should be lowering the mean or the peak GH loads. The controversy is caused by the lack of consensus on what is more damaging to the tissue, less frequent high forces (with longer recovery periods) or persistent high frequency lower forces. According to the studies on animal models of muscle damage and studies documenting repetitive workplace injuries, even low-load movement sustained for an extended period, has a great potential to cause overuse damage to the muscle and ligament tissue [38]. The highest injury-inducing potential lies in the high-repetition and high-load tasks but the exact relationship between the dose of mechanical load and tissue response is unknown.

### Effect of practice on individual muscle force

The highest mean relative forces and highest power production at the pre- and the post-test were generated by a pair of antagonist muscles: the triceps and the bices muscle. Those two muscles showed to generate power interchangeably during the majority of the push phase [39] and remain among the largest contributors to the power generation throughout the propulsive cycle independent of the simulated propulsion technique [36]. Large contribution of the triceps muscle is in accordance with the study of Vegter et al. [5] who also found that triceps was the biggest power producer in the very initial stages of practice in wheelchair propulsion and with a study of Slowik et al. [40] who found the triceps muscle among the primary contributors to the propulsive cycle, independent of the hand pattern during the recovery phase. It is however striking that the magnitude of the peak relative force production of the triceps in the current study exceeded 50%, both at the pre- and the post-test, while in the study of Vegter et al., it oscillated around 15%. Another study reported triceps force production of 25% [1]. The second biggest contributor was the biceps muscle exerting approximately 40% of the attainable force at both testing occasions and largely contributing to the power production. Contribution of the biceps is higher in the current study than in previous literature. Veeger et al. [1] found peak relative force of biceps of just under 25% in experienced wheelchair users and Vegter et al. a contribution of approx. 15% [5]. The notably high contributions of biceps and triceps muscles in the present study may be related to the quite large differences in push frequency between the studies. Vegter et al [5] (>55 push/min) and Veeger et al [1] (96 pushes/min) reported a higher frequency compared to our study (36 pushes/min). Both experiments were performed at a very similar velocity and power output as the current study. As our participants lowered the push frequency to 36 pushes/min, more muscle power had to be exerted during the push to maintain the treadmill velocity. This explanation is supported by a previous study which used forward dynamics simulation and found that simulating minimizing push frequency and maximizing contact angle both result in large increases in power production of biceps and triceps muscles during the push phase [36].

The contributions of biceps and triceps are followed by large power and relative force production of pectoralis major and the following rotator cuff muscles: supraspinatus, infraspinatus and subscapularis. High contribution of those muscles during the push phase is in accordance with other studies with experienced wheelchair users [1,41] and able-bodied persons [5,33]. Supraspinatus produced relative force of almost 40% at the post-test which is comparable to the values found in experienced wheelchair users [1]. High load on all rotator cuff muscles, but especially supraspinatus is concerning. Supraspinatus muscle, because of its relatively small size, and anatomical location, running through the narrow subacromial space, is vulnerable to overload and impingement [42]. Finally, the contribution of the anterior part of the deltoideus was very low in the able-bodied participants in the current study. This is agreement with a previous study which used the same musculoskeletal model and a very comparable participant group [5] but contradicts other studies which reported it to be a main contributor [39,41].

### Advantages and limitations

The primary advantage of this observational single group pre-post experiment is that the changes in shoulder load were investigated over a longer practice period, excluding the very initial stages that have been documented before [5]. Including able-bodied participants is a useful method to preliminary examine the kinetic and dynamic changes occurring during the learning process. Firstly, because the participant have no experience at the onset of the study. Secondly, because confounders that are present in actual wheelchair users such as sitting balance, presence of pain or limited muscle function cannot influence the acquired results. However, to be able to fully understand the way propulsion technique relates to shoulder pain, actual wheelchair users should be investigated in future studies. Although 7 participants is not a very unusual number when compared with other modeling studies [1,5], we recommend to include more participants in the future. Power analysis calculation (G*Power, α = 0.05; Power = 0.80; [43]) indicated that 11 participants would be necessary to reach a significant decrease in the mean glenohumeral reaction force per cycle. In order to make sure that the test conditions were standardized for all participants, no individual fitting was provided within the user-wheelchair interface. Although this could initially have some influence on the propulsion technique, it should be considered that the wheelchair remained unchanged between the pre- and the post-test. All observed changes were therefore a result of practice. Although within-subject comparisons remain valid, across-subject comparisons may be confounded by the fixed dimensions of the experimental wheelchair in the context of individual anthropometric differences. Lastly, DSEM is a non-personalized musculoskeletal model. This means that for the well-trained individuals and athletes the relative loads could be lower as their muscles are habituated to handle higher loads.

### Future directions

Future studies should include actual wheelchair users, preferably with various levels of experience and various lesion levels, in order to further explore the relationship between wheelchair propulsion technique and upper extremity concerns. Investigation should take place as early as possible in the learning process since recent evidence suggests that shoulder pain develops already in the early stages of rehabilitation [2]. Future research should attempt to determine how the quantified changes in shoulder load influence the actual damage to the soft tissue. Including a higher number of participants could allow forming clusters of individuals with similar propulsion characteristics, which could explain various trajectories of overuse injury development. Moreover, larger participant group could help to determine what the underlying biological patterns are that drive the optimization in cyclic wheelchair exercise.

## Conclusions

Changes in the propulsion technique found in this pre-post single group study are in agreement with other low-intensity wheelchair practice studies and values at the post-test resemble the values in actual wheelchair users. Despite developing a uniform propulsion technique, the kinematic solution to the task varied in magnitude and direction between the participants. In contrast to the rather variable kinematic output, participants exhibited two glenohumeral reaction force distribution patterns during the propulsive cycle. Individuals, who developed high force at the onset of the push, increased both peak and mean glenohumeral forces. Participants, who distributed the force more evenly over the propulsive cycle, lowered both peak and mean glenohumeral force. This study provides preliminary insights on the possible relation between the changes in wheelchair propulsion technique and shoulder load taking place after low-intensity practice. This knowledge should be extended in the future by investigating actual wheelchair users with various levels of experience.

## Supporting information

S1 Data Complete data set. (XLSX)
